# Design of a Dual-Band Low-Noise Amplifier with a Novel Matching Structure

**DOI:** 10.3390/mi16080938

**Published:** 2025-08-15

**Authors:** Mingwen Zhang, Zhiqun Cheng, Tingwei Gong, Bangjie Zheng, Zhiwei Zhang, Xuefei Xuan

**Affiliations:** 1 School of Electronics and Information, Hangzhou Dianzi University, Hangzhou 310018, China; mwzhang@wuyiu.edu.cn (M.Z.); gtw1016032898@163.com (T.G.); jackzheng@hdu.edu.cn (B.Z.); zhiwei_zhang@hdu.edu.cn (Z.Z.); xuanxuefei2022@163.com (X.X.); 2 School of Mechanical and Electrical Engineering, Wuyi University, Wuyishan 354300, China; 3 The Key Laboratory for Agricultural Machinery Intelligent Control and Manufacturing of Fujian Education Institutions, Wuyishan 354300, China; 4 School of Information Engineering, Xinjiang Institute of Technology, Akesu 843100, China

**Keywords:** complex impedance transformation, dual-band LNA, parallel-coupled lines, transmission line

## Abstract

This paper proposes a method for designing a dual-band low-noise amplifier (DB-LNA) using a new improved complex impedance dual-band transformer (IDBT). This complex IDBT is composed of parallel-coupled lines and two sections of series microstrip lines. The parallel-coupled lines are used to complete the transformation from complex impedances at two different frequencies to a pair of conjugate complex impedances, meanwhile eliminating the need for DC blocking capacitors. The transformation to real impedances is achieved by series microstrip lines at dual frequency points. A single-stage DB-LNA was designed using the BFP840ESD transistor in combination with the proposed IDBT. The fabrication and testing of the Printed Circuit Board (PCB) were then completed. The measured results of the proposed 2.4/5.5 GHz DB-LNA show an S21 parameter of 20.3/14.7 dB, an S11 of −29.8/−20.3 dB, an S22 of −15.2/−16.4 dB, and a noise figure (NF) of 1.6/1.6 dB. The whole DB-LNA has a simple structure, low cost, and excellent performance and is easy to tune.

## 1. Introduction

With the advancement of wireless technology, wireless local area networks (WLANs) are playing an increasingly crucial role in people’s lives. Concurrently, as WLAN standards continue to proliferate, multi-band, multi-functional, and cost-effective WLAN devices are demanded to enhance both device integration and user convenience. Meanwhile, for the convenience of communication device usage, 5 GHz band WLANs must be back-ward-compatible with the 2.4 GHz band [1]. WLAN communication performance is significantly influenced by the LNA in the RF front-end. Therefore, LNAs are required by dual-band WLANs to support simultaneous dual-band operation.

**Figure 1:**
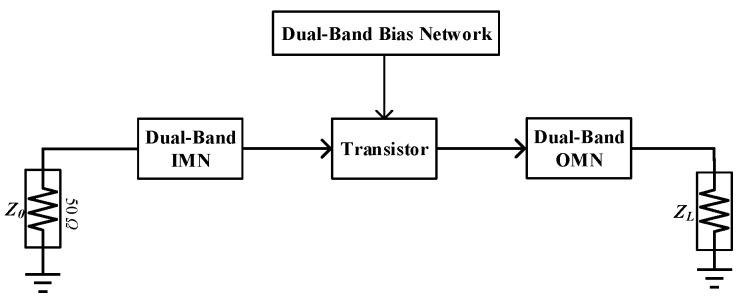
A typical DB-LNA structure block diagram.

Over the years, numerous DB-LNA design structures have been proposed, including parallel [1], switch-tunable [2], broadband [3], and concurrent configurations [4,5,6,7,8,9,10,11]. Among these, the concurrent DB-LNA topology (illustrated in Figure 1) is composed of an input matching network (IMN), an output matching network (OMN), a dual-band bias network, and a transistor. This configuration offers key advantages, such as simultaneous operation across two distinct frequency bands, enabling effective amplification of in-band signals while suppressing out-of-band interference. However, concurrent DB-LNAs also present significant challenges: (1) complex matching network design, requiring precise dual-frequency impedance matching within a single channel; (2) degraded in-band noise performance and limited gain in target frequency bands; and (3) excessive discrete components, where parasitic effects from externally soldered DC-blocking capacitors compromise IMN/OMN performance. The core challenge lies in the matching circuit design. Early approaches leveraged traditional microstrip line theory [12,13,14,15], while later advancements incorporated parallel-coupled lines [16,17,18,19]. Recent systematic studies [12,13,14,15,16,17,18,19] have focused on IDBTs as a key innovation, conducting multidimensional optimizations to address these limitations.

Therefore, a novel IDBT structure based on parallel-coupled lines and microstrip lines is proposed in this paper. The DC-blocking capacitor is eliminated by the inherent characteristic of parallel-coupled lines in this IDBT structure, thus reducing parasitic parameter effects. Meanwhile, no microstrip stubs are included, which not only simplifies the overall design but also contributes to the realization of a more compact transformer structure. The theoretical analysis and detailed parameter calculation of this transformer structure have been comprehensively conducted. In this study, the design and measurement of a DB-LNA are implemented using both the proposed IDBT structure and discrete components, specifically the Infineon BFP840ESD transistor. The amplifier is fabricated through Rogers 4350B PCB board-level processing, with comprehensive experimental characterization subsequently performed.

## 2. Design Theory and Analysis

This section consists of the following parts:

1. First of all, the IDBT proposed for adoption in this paper will be introduced as a whole.
2. Secondly, the relevant theory of parallel-coupled lines will be introduced to theoretically prove the feasibility of the design.
3. Thirdly, the calculation method of the two-section series microstrip lines in Part II will be introduced.
4. Finally, the implementation method of low NF in the DB-LNA will be analyzed.

### 2.1. Complex IDBT

**Figure 2:**
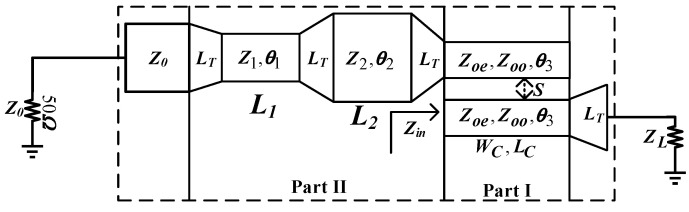
The schematic diagram of the complex IDBT.

The schematic diagram of the new complex IDBT used in this article is shown in Figure 2. The structure consists of Part I and Part II. Part I includes a section of parallel-coupled lines with electrical length *θ*_3_ and odd–even characteristic impedance *Z_oo_* and *Z_oe_*. Part II includes two sections of series microstrip transmission lines with characteristic impedances *Z*_1_ and *Z*_2_, respectively, and electrical lengths *θ*_1_ and *θ*_2_. All connections in the entire structure are realized using gradient microstrip lines of length *L_T_*. In Figure 2, *Z_L_* represents the complex impedance from the transistor gate or drain, denoted as *Z_L_*_1_ and *Z_L_*_2_ at two different frequencies *f*_1_ and *f*_2_ (*f*_1_ < *f*_2_). It is assumed that *Z_L_*_1_ *= R_L_*_1_ *+ jX_L_*_1_ @ *f*_1_ and *Z_L_*_2_ = *R_L_*_2_ + *jX_L_*_2_ @ *f*_2_ will have different complex values at different frequencies in most cases, so *R_L_*_1_ and *R_L_*_2_, and *X_L_*_1_ and *X_L_*_2_ are not equal and are independent of frequency. Part I transforms *Z_L_*_1_ and *Z_L_*_2_ at frequencies *f*_1_ and *f*_2_ into a pair of conjugate impedance values *Z_in_*_1_ and *Z_in_*_2_ through parallel-coupled lines. Part II converts the conjugate *Z_in_*_1_ and *Z_in_*_2_ into *Z*_0_ at both frequencies through two sections of series microstrip transmission lines.

### 2.2. Parallel-Coupled Line Theory

**Figure 3:**
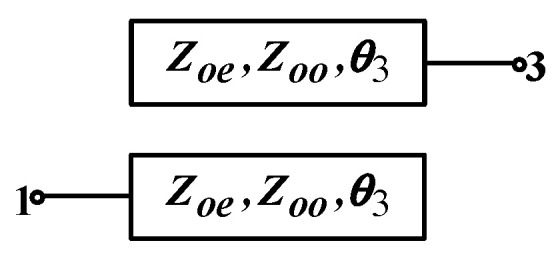
The dual-port coupled-line structure adopted in this paper.

As shown in Figure 3, when ports 2 and 4 of the structure are open-circuited, the parallel-coupled line structure is equivalent to a two-port device, and the corresponding impedance matrix equation can be represented by Equation (1):

(1) $${\{}_{V_{3}\;=\;Z_{31}I_{1}\;+\;Z_{33}I_{3}}^{V_{1}\;=\;Z_{11}I_{1}\;+\;Z_{13}I_{3}}$$

Then its ABCD matrix is given by Equation (2):

(2) [DBCA]=2j(Zoe−Zoo)cscθ2(Zoe+Zoo)cotθ2(Zoe−Zoo)cscθ2    (Zoe+Zoo)cotθ2(Zoe−Zoo)cscθ2j2Zoe2+Zoo2−2ZoeZoo(cos2θ2+csc2θ2)(Zoe−Zoo)cscθ2

In Figure 2, after the transformation by the Part I parallel-coupled line, the conjugate impedance obtained can be expressed as Equation (3):

(3) $$Z_{in2}=R_{in1}-jX_{in1}=Z_{in1}^{*}$$

Here, the asterisk (*) in Equation (3) represents the conjugate complex impedance.

The parallel-coupled line can simultaneously transform *Z_L_*_1_ and *Z_L_*_2_ at two frequencies into *Z_in_*_1_ and *Z_in_*_2_, which are conjugate to each other. The relevant calculation process is presented in Equations (4a) and (4b) as follows:

(4a) $$R_{in1}=\frac{A_{1}(R_{L1}+jX_{L1})+B_{1}}{C_{1}(R_{L1}+jX_{L1})+D_{1}}$$

(4b) $$R_{in2}=\frac{A_{2}(R_{L2}+jX_{L2})+B_{2}}{C_{2}(R_{L2}+jX_{L2})+D_{2}}$$

Then, combining Equations (2) and (4), we can obtain Equations (5a) and (5b):

(5a) $$(Z_{oe}+Z_{oo})(R_{in1}-R_{L1})\cot\theta =2R_{in1}X_{L1}$$

(5b) $$\begin{array}{l} 2(Z_{oe}+Z_{oo})\cot\theta X_{L1}+[Z_{oe}^{2}+Z_{oo}^{2}\\ -Z_{oe}Z_{oo}(\cos_{\theta }^{2}+\csc_{\theta }^{2})]=4R_{in1}R_{L1} \end{array}$$

At the two frequencies *f*_1_ and ***f*_2_**, by simultaneously solving Equation (5), we can obtain a set of simple analytical design equations for the even-mode and odd-mode characteristic impedances of the parallel-coupled line, as shown in Equation (6):

(6a) $$Z_{oo}=\frac{R_{in1}X_{L1}\tan\theta_{3}}{R_{in1}-R_{L1}}(1\pm \sqrt{1-\frac{2}{1+\cos_{\theta_{3}}^{2}+\csc_{\theta_{3}}^{2}}})$$

(6b) $$Z_{oe}=\frac{2R_{in1}X_{L1}\tan\theta_{3}}{\left(R_{in1}-R_{L1}\right)}-Z_{oo}$$

In Equation (6), “±” needs to be flexibly selected according to the actual situation.

Here, the electrical length of the parallel-coupled line can be expressed as Equation (7):

(7) $$\theta_{3}\;|\;f_{1}=\frac{\pi }{1+p}$$

In Equation (7), *p = f*_2_/*f*_1_. Assume (*f*_1_ *< f*_2_). In this way, based on Equations (6) and (7), the even-mode and odd-mode impedances and the electrical length related to the parallel-coupled lines can be obtained.

### 2.3. Design of Two-Section Microstrip Transmission Lines in Part II

In Figure 2, the parameters of the parallel-coupled lines are determined, transforming the load impedance into a pair of conjugate complex impedance values, which are to be transformed into the desired impedance by two sections of microstrip lines in Part II. Thus, we can obtain the following Expression (8):

(8a) $$Z_{0}=Z_{1}\frac{Z_{ina}+jZ_{1}\tan(\theta_{1})}{Z_{1}+jZ_{ina}\tan(\theta_{1})}$$

(8b) $$Z_{ina}=Z_{2}\frac{Z_{in}+jZ_{2}\tan(\theta_{2})}{Z_{2}+jZ_{in}\tan(\theta_{2})}$$

Assuming that at frequency *f*_1_, *Z_in_*_1_ = *R_in_*_1_ + *jX_in_*_1_, Equation (8) can be rewritten as follows:

(9a) $$\begin{array}{l} Z_{0}X_{in1}(Z_{1}\tan(\theta_{2}\;|\;f_{2})+Z_{2}\tan(\theta_{1}\;|\;f_{1}))+Z_{1}Z_{2}R_{in1}\\ -Z_{1}Z_{2}Z_{0}-\tan(\theta_{1}\;|\;f_{1})\tan(\theta_{2}\;|\;f_{2})(R_{in1}Z_{1}^{2}-Z_{0}Z_{2}^{2})=0 \end{array}$$

(9b) $$\begin{array}{l} Z_{0}R_{in1}(Z_{1}\tan(\theta_{2}\;|\;f_{2})+Z_{2}\tan(\theta_{1}\;|\;f_{1}))\\ -Z_{1}^{2}Z_{2}\tan(\theta_{1}\;|\;f_{1})-Z_{1}Z_{2}^{2}\tan(\theta_{2}\;|\;f_{2})-Z_{1}Z_{2}Z_{in1}\\ +X_{in1}\tan(\theta_{1}\;|\;f_{1})\tan(\theta_{2}\;|\;f_{2})=0 \end{array}$$

Expressions (9) correspond to two frequencies, resulting in four variables for the two equations. Generally, such equations can only be solved by numerical methods or optimization algorithms. Due to the conjugate relationship of *Z_in_*, similar to [13,14], the following Equation (10) can be obtained:

(10a) $$\tan(\theta_{1}\;|\;f_{1})\pm \tan(\theta_{1}\;|\;f_{1})=0$$

(10b) $$\tan(\theta_{2}\;|\;f_{2})\pm \tan(\theta_{2}\;|\;f_{2})=0$$

Further assume that *θ = βl*. Substituting this into Equation (10), we can obtain the roots of *l_1_* and *l*_2_. Similarly to the analysis in [14], considering the area, we choose Equation (11):

(11) $$l_{1}=l_{2}=\frac{\pi }{\beta_{a}+\beta_{b}}=l$$

Let us further define *a = tan*(*β_a_l*). Considering the requirement for a small area, in the minimum case, the following Expression (12) can be listed:

(12) $$Z_{1}^{4}+bZ_{1}^{3}+cZ_{1}^{2}+dZ_{1}+e=0$$

(12a) $$b=\frac{2aR_{0}X_{in}}{R_{0}-R_{in}}$$

(12b) $$c=\frac{R_{0}R_{in}(X_{in}^{2}-{\left(R_{0}-R_{in}\right)}^{2})-X_{in}^{2}R_{0}^{2}{\left(1+a^{2}\right)}^{2}}{a^{2}R_{in}(R_{in}-R_{0})}$$

(12c) $$d=\frac{2aR_{0}^{3}X_{in}}{R_{in}-R_{0}}$$

(12d) $$e=\frac{R_{0}^{3}(R_{in}^{2}+X_{in}^{2}-R_{0}R_{in})}{R_{0}-R_{in}}$$

Based on Equation (12), it is easy to solve this fourth-order equation, thereby obtaining the value of *Z*_1_. Then, according to Formula (9), the impedance values and electrical lengths of the two-section microstrip transmission lines in Part II can be determined.

### 2.4. Implementation of Low NF in the DB-LNA

The basic definition of the NF is the ratio of the signal-to-noise ratio at the amplifier input to that at the output. The NF is an important indicator for LNAs. For a single-stage amplifier, the NF [3] can be expressed as Equation (13):

(13) $$F=F_{\min}+\frac{4r_{n}\left|\Gamma_{s}-\Gamma_{opt}\right|}{(1-{\left|\Gamma_{s}\right|}^{2}){\left|1+\Gamma_{opt}\right|}^{2}}$$

Here, *F_min_* is the minimum NF of the transistor, Г*_opt_* is the source reflection coefficient for obtaining the minimum NF, and *r_n_* is the normalized noise resistance. These three parameters are all noise parameters of the transistor. Г*_s_* is the actual source reflection coefficient of the amplifier; *F_min_* is a function of the transistor operating current and frequency, and for each *F_min_* there is a corresponding Г*_op_*_t_. For LNAs, the role of the IMN is to achieve equal-value matching between the source impedance and the input impedance corresponding to the minimum NF of the transistor. The OMN needs to achieve conjugate matching between the output impedance and the load impedance to achieve high gain, which is also a relatively difficult goal. The dual-band IMN proposed in this paper achieves equal-value matching at two frequency points simultaneously to obtain a low NF, while the OMN realizes conjugate matching at both frequencies concurrently to achieve high gain.

**Figure 4:**
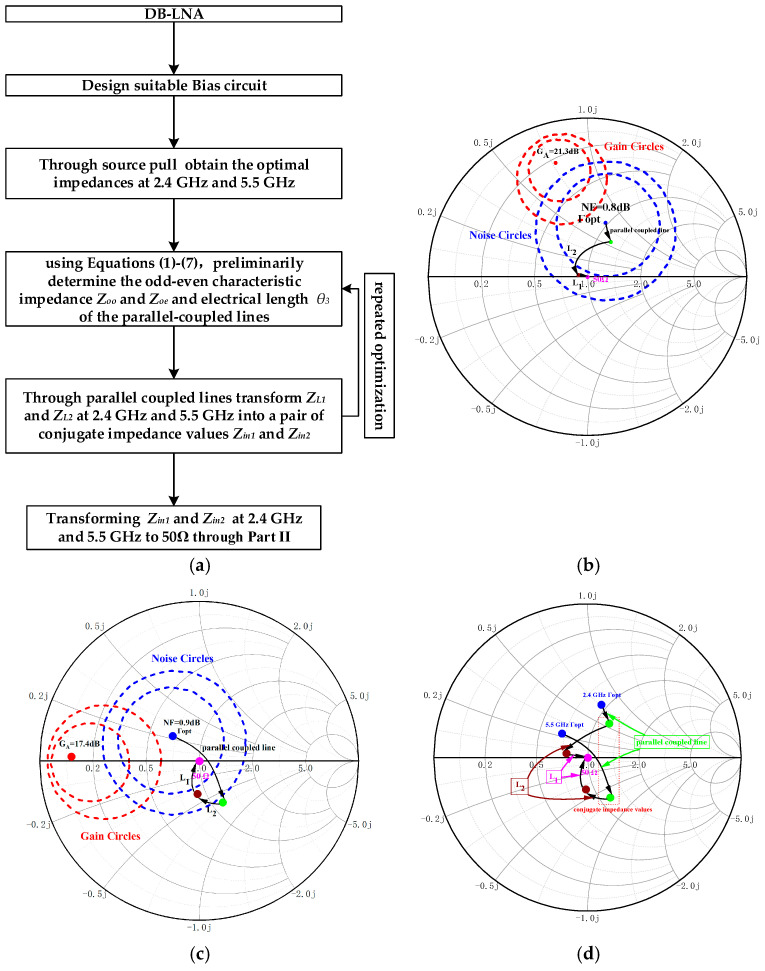
Main process flowchart of the proposed method when designing the DB-LNA (**a**). Available *G_A_* and NF circles at 2.4 GHz (**b**) and 5.5 GHz (**c**), with the input matching reflection coefficient set to 50 Ω for the minimum NF. Dual-frequency matching process diagram (**d**).

The above approach is summarized in the flowchart in Figure 4a. And Figure 4 plots the simulated available gain (*G_A_*) and NF circles at 2.4 GHz (b) and 5.5 GHz (c). At 2.4 GHz (a), the Г*_opt_* is matched to 0.1∠−124° through the parallel-coupled line and then matched to 0.07∠175° through *L*_2_ and matched to 50 Ω through *L*_1_ to achieve a minimum NF = 0.8 dB, with *G_A_* = 20.9 dB. Similarly, at 5.5 GHz, the Г*_opt_* is matched to 50 Ω through the parallel-coupled line and *L_2_* and *L_1_* to achieve a minimum NF = 0.9 dB, with *G_A_* = 16.4 dB. Figure 4d depicts the process of dual-frequency impedance matching. Similarly to input matching, the OMN conjugately matches the value of Ga_opt to 50 Ω at 2.4 GHz and 5.5 GHz, respectively, aiming to achieve maximum power transfer.

## 3. Implementation and Measurement

**Figure 5:**
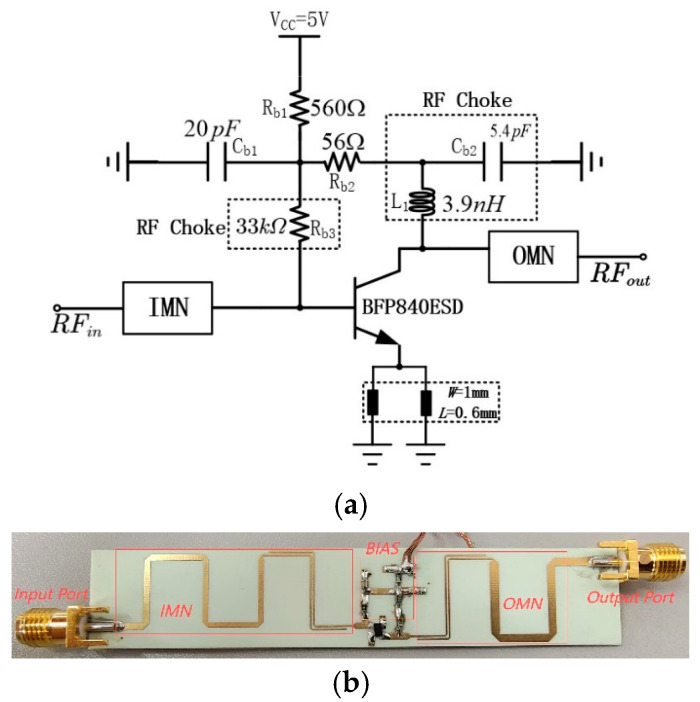
(**a**) Schematic diagram of DB-LNA and (**b**) the fabricated DB-LNA.

Based on the above theoretical analysis, the 2.4/5.5 GHz DB-LNA has been fabricated. Based on the Infineon heterojunction bipolar transistor BFP840ESD, the IDBT analyzed in Part II is utilized as the IMN and OMN structures, and a bias circuit is designed for it. Figure 5 shows the complete circuit diagram (a) and the physical picture (b). Rogers 4350B board material is used, with a thickness of 0.762 mm, a dielectric constant of 3.66, and a dielectric loss tangent of 0.0037. The resistors, capacitors, and inductors are from Panasonic’s ERJ3GEYJ series and Murata’s GRM1885C1H series and LQG18HN series, respectively. The fabricated PCB with dimensions of 85 mm × 16 mm is physically demonstrated in Figure 5b; the bias circuit, IMN, OMN, etc., are all marked.

**Figure 6:**
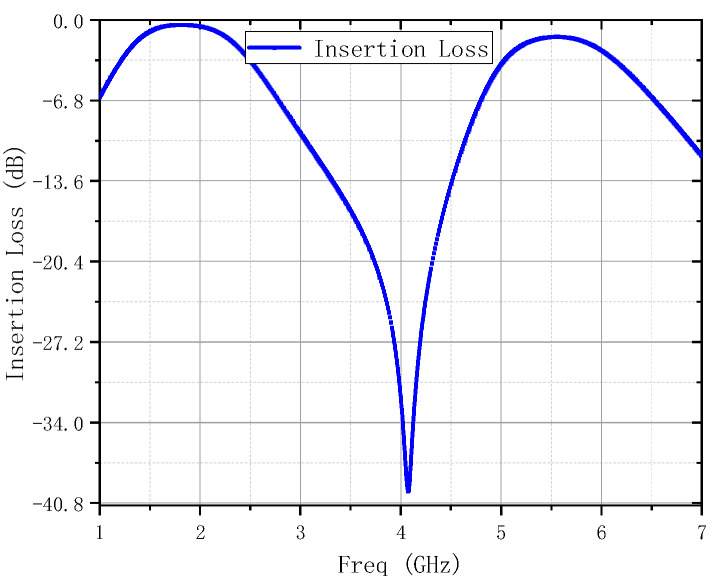
EM simulated insertion loss of the IMN.

**Figure 7:**
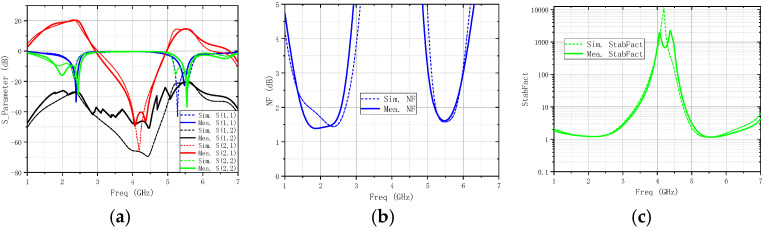
Simulated and measured performance: (**a**) S-parameter, (**b**) NF, and (**c**) StabFact.

**Figure 8:**
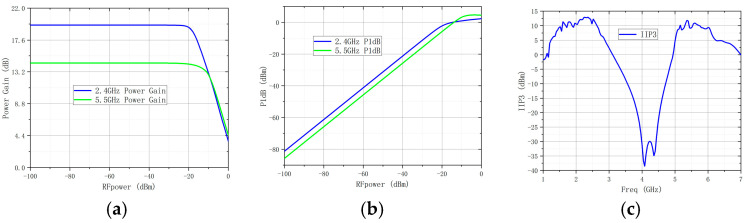
Measured linearity performance: (**a**) power gain, (**b**) P1dB, and (**c**) IIP3 vs. frequency.

As can be seen from Figure 6, the EM simulated insertion losses of the IMN are 2.5 dB and 1.4 dB at 2.4 GHz and 5.5 GHz. In the measurement, the S-parameters of this DB- LNA were measured by a 3656B network analyzer (Siglent SNA5000A), and its noise was analyzed by a spectrum analyzer (Siglent SSA5000A). A vector network analyzer (N5244A) and a power sensor (U2021XA) were utilized to measure the linearity. The simulated and measured performances are shown in Figure 7, from which it can be seen that it can cover frequency ranges of 2.33–2.46 GHz and 5.43–5.58 GHz. Meanwhile, the corresponding S11 values are −29.8 dB and −20.3 dB, the corresponding S22 values are −15.2 dB and −16.4 dB, the corresponding S21 values are 20.3 dB and 14.7 dB, and the corresponding NF values are 1.6 dB and 1.6 dB. In the operating frequency bands, the stability factor is greater than 1.1, indicating that it is absolutely stable within the operating frequency bands. The linearity of the DB-LNA is checked by feeding one tone with RFpower from −100 dBm to 0 dBm, with the 2.4 GHz and 5.5 GHz power gain and P1dB illustrated in Figure 8a,b. The dependence of IIP3 on frequency is also depicted in Figure 8c. Due to the parallel-coupled line configuration, the developed LNA exhibits good linearity within the design frequency band.

The proposed DB-LNA in this work was designed, simulated, and measured. For the actual design, the measured results are shown in Table 1. Due to the limitation of the technique and manufacture, some deviations exist between the simulation and measurement.

## 4. Conclusions

In this paper, a low-cost, board-level DB-LNA featuring a novel matching structure with simplified architecture is presented. A hybrid matching network combining parallel-coupled lines and microstrip lines is utilized in the design, and the inherent DC-blocking characteristics of the coupled lines are leveraged to eliminate external blocking capacitors. Reliability is improved by avoiding solder joint effects and parasitic parameters through this approach. Measured results show excellent performance, with dual-band operation at 2.4/5.5 GHz, S21 > 14 dB, and NF < 1.7 dB. The proposed DB-LNA exhibits strong potential for WLAN applications.

## Figures and Tables

**Table 1:** Performance comparison of DB-LNAs.

Ref.	Frequency (GHz)	S11 (dB)	S22 (dB)	S21 (dB)	NF (dB)	Size (mm^2^)	Power (mW)	Technology
[4]	2.4	−11.3	−24.6	33.84	0.946	-	-	GaAs MMIC
	5.75	−17.4	−11.1	20	0.493			
[5]	2.4	−10	−8	20	2.2	1.5 × 1	37.8	GaAs MMIC
	5	−7	−6	15	2.0			
[6]	2.45	−20	-	22	1.5	30 × 30	7.5	HMIC
	5.2	−21	-	12	1.6			
[7]	2.3–2.5	−8.5	-	3–12.2	0.5–5	55 × 60	41.25	HMIC
	4.2–4.6	−15	-	9.5–12.9	2.5–5			
[8]	2.44	−10.5	-	7.15	4.34	-	35.1	HMIC
	5.25	−15.9	-	7.8	4.69			
[9]	2.4	−25	-	11.6	3.96	120 × 34	56	HMIC
	5.7	−12	-	8.9	2.89			
This Work	2.33–2.46	−29.8	−15.2	20.3	1.6	16 × 85	39.3	HMIC
	5.43–5.58	−20.3	−16.4	14.7	1.6			

## Data Availability

The data presented in this work are available within the article.

## References

[1] Schmidt A., Catala S. (2001). A Universal Dual Band LNA Implementation in SiGe Technology for Wireless Applications. IEEE J. Solid-State Circuits.

[2] Yu X., Neihart N.M. (2013). Analysis and Design of a Reconfigurable Multimode Low-Noise Amplifier Utilizing a Multitap Transformer. IEEE Trans. Micro-Wave Theory Tech..

[3] Hu R. (2004). An 8–20-GHz Wide-Band LNA Design and the Analysis of Its Input Matching Mechanism. IEEE Microw. Wirel. Compon. Lett..

[4] Ahmad A., Othman A.R., Hamidon A.H., Pongot K. (2016). A Low Noise Dual-Band Cascaded LNA with Notch Filtering Network for IEEE 802.11b/g/a/n Wireless Applications. ARPN J. Eng. Appl. Sci..

[5] Hsiao Y.-C., Meng C., Yang C. (2016). Design Optimization of Single-/Dual-Band FET LNAs Using Noise Transformation Matrix. IEEE Trans. Micro-Wave Theory Tech..

[6] Djoumessi E.E., Wu K. Dual-Band Low-Noise Amplifier Using Step-Impedance Resonator (SIR) Technique for Wireless System Applications. Proceedings of the 39th European Microwave Conference.

[7] Kumar A., Pathak N.P. Reconfigurable Concurrent Dual-Band Low Noise Amplifier for Non-invasive Vital Sign Detection Applications. Proceedings of the 2014 International Conference on Advances in Computing, Communications and Informatics.

[8] Iyer B., Pathak N.P. (2014). A Concurrent Dual-Band LNA for Noninvasive Vital Sign Detection System. Microw. Opt. Technol. Lett..

[9] Kumar A., Pathak N.P. Coupled Stepped-Impedance Resonator (CSIR) Based Concurrent Dual Band Filtering LNA for Wireless Applications. Proceedings of the 2015 IEEE MTT-S International Microwave and RF Conference (IMaRC).

[10] Lee J., Nguyen C. (2018). A K-/Ka band Concurrent Dual-Band Low-Noise Amplifier Employing a Feedback Notch Technique with Simultaneous Passband Gain and Stopband Rejection Control. Microw. Opt. Technol. Lett..

[11] Gupta M.P., Kumar S., Caroline B.E., Song H., Kumar V., Gorre P. (2023). A 0.15 μm GaN HEMT Device to Circuit Approach Towards Dual-Band Ultra-Low Noise Amplifier Using Defected Ground Bias Technique. Int. J. Electron. Commun..

[12] Nikravan M.A., Atlasbaf Z. (2011). T-Section Dual-Band Impedance Transformer for Frequency-Dependent Complex Impedance Loads. Electron. Lett..

[13] Manoochehri O., Asoodeh A., Forooraghi K. (2015). II-Model Dual-Band Impedance Transformer for Unequal Complex Impedance Loads. IEEE Microw. Wirel. Compon. Lett..

[14] Zheng X., Liu Y., Li S., Yu C., Wang Z., Li J. (2012). A Dual-Band Impedance Transformer Using PI-Section Structure for Frequency-Dependent Complex Loads. Prog. Electromagn. Res..

[15] Chuang M.-L. (2014). Analytical Design of Dual-Band Impedance Transformer with Additional Transmission Zero. IET Microw. Antennas Propag..

[16] Chen M.-G., Hou T.-B., Tang C.-W. (2012). Design of Planar Complex Impedance Transformers with the Modified Coupled Line. IEEE Trans. Compon. Packag. Manuf. Technol..

[17] Fang S.J., Jia X. (2020). Design of Compact Coupled-Line Complex Impedance Transformers with the Series Susceptance Component. IEEE Trans. Circuits Syst. II Express Briefs.

[18] Guo Y.Q., Liu H.M. Modification of Susceptance Component Loaded Coupled Line Complex Impedance Transformer. Proceedings of the 2020 Cross Strait Radio Science & Wireless Technology Conference (CSRSWTC).

[19] Wu Y., Sun W., Leung S.-W., Diao Y., Chan K.-H. (2013). A Novel Compact Dual-Frequency Coupled-Line Transformer with Simple Analytical Design Equations for Frequency-Dependent Complex Load Impedance. Prog. Electromagn. Res..

